# Taurine Alleviates Intestinal Injury by Mediating Tight Junction Barriers in Diquat-Challenged Piglet Models

**DOI:** 10.3389/fphys.2020.00449

**Published:** 2020-05-28

**Authors:** Chaoyue Wen, Qiuping Guo, Wenlong Wang, Yehui Duan, Lingyu Zhang, Jianzhong Li, Shanping He, Wen Chen, Fengna Li

**Affiliations:** ^1^Hunan Provincial Key Laboratory of Animal Nutritional Physiology and Metabolic Process, Changsha, China; ^2^Key Laboratory of Agro-Ecological Processes in Subtropical Region, Institute of Subtropical Agriculture, Chinese Academy of Sciences, Changsha, China; ^3^Hunan Provincial Engineering Research Center for Healthy Livestock and Poultry Production, Changsha, China; ^4^National Engineering Laboratory for Pollution Control and Waste Utilization in Livestock and Poultry Production, Changsha, China; ^5^Scientific Observing and Experimental Station of Animal Nutrition and Feed Science in South-Central, Ministry of Agriculture, Changsha, China; ^6^Laboratory of Animal Nutrition and Human Health, College of Life Science, Hunan Normal University, Changsha, China; ^7^Hunan International Joint Laboratory of Animal Intestinal Ecology and Health, College of Life Science, Hunan Normal University, Changsha, China; ^8^Hunan Provincial Key Laboratory of Animal Intestinal Function and Regulation, College of Life Science, Hunan Normal University, Changsha, China; ^9^University of Chinese Academy of Sciences, Beijing, China

**Keywords:** immune response, oxidative stress, taurine, tight junction barrier, weaned piglets

## Abstract

**Background:** Intestinal barrier contributes as an important role in maintaining intestinal homeostasis. Oxidative stress can cause critical damages in intestinal integrity of animals.

**Objectives:** This study was conducted to investigate the alleviated effect of taurine against small intestine (duodenum, jejunum, ileum) injury induced by oxidative stress.

**Methods:** The piglet model of diquat-induced oxidative stress was employed. In addition, analysis of intestinal morphology, reverse transcription PCR (RT-PCR), and Western blot were used in this study.

**Results:** Compared with the control group (CON), diquat-induced oxidative stress triggers immune response; the content of immunoglobulin M (IgM) and immunoglobulin G (IgG) was significantly changed, but 0.60% taurine supplementation could restore the level of serum immunoglobulin. Oxidative stress induces serious damage in intestinal morphology structure and tight junction barrier. Compared with the CON, the villus height of intestine was significantly decreased, the crypt depth and villus height/crypt depth (V/C) were also decreased, and 0.60% taurine supplementation could restore impaired morphology and even improve crypt depth and V/C of the jejunum and ileum. Compared with the CON, oxidative stress markedly increased the messenger RNA (mRNA) expression level of *claudin-1* and *occludin* in the duodenum, and the value of *occludin* was significantly decreased in the jejunum of the diquat group (DIQ). Relative to the DIQ, 0.60% taurine supplementation increased the mRNA expression level of *claudin-1*, *occludin*, and *ZO-1* in the ileum. Compared with the CON, the expression of claudin-1 protein was significantly upregulated, and occludin and ZO-1 protein were both downregulated in the small intestine of DIQ.

**Conclusion:** Taurine exerts protective effects by regulating immune response and restores the intestinal tight junction barrier when piglets suffer from oxidative stress.

## Introduction

Oxidative stress occurs throughout animal life, with many factors contributing to the induction of oxidative stress, such as birth, weaning, changes in food sources, and transportation (Gu et al., 2011; Campbell et al., 2013; Yin et al., 2014; Luo et al., 2016; Guo et al., 2019). Weaning induces both acute and long-lasting changes in the intestines of piglets, including the shortening of villus height and increasing crypt depth (Boudry et al., 2004), as well as reducing digestive enzyme activities (Pluske et al., 1997). After weaning for 3 days, the jejunal antioxidant-related genes (Cu–Zn superoxide dismutase, Mn superoxide dismutase, glutathione peroxidase 1, and glutathione peroxidase 4) of piglets are significantly decreased (Yin et al., 2014), and cytokines’ mRNA expression level [tumor necrosis factor alpha (TNF-α), interferon gamma (IFN-γ), and interleukin (IL-6)] are significantly increased; jejunal epithelial permeability are markedly raised (Hu et al., 2013). Diquat is widely used to induce oxidative stress in intestine of piglets (Wang et al., 2012; Yuan et al., 2017; Cao et al., 2018). Diquat-induced oxidative stress has a negative effect on jejunal and ileal morphology (Wang et al., 2012; Yuan et al., 2017), and it could increase the histopathological grading of the jejunum, ileum, and colon (Yuan et al., 2017); the activities of antioxidant enzymes (superoxide dismutase, glutathione peroxidase) and the permeability of jejunal epithelium significantly decrease (Cao et al., 2018). Thus, the weaning period is the time in which piglets experience an increased prevalence of gastrointestinal dysfunctions, which in turn reduces growth performance in pigs and subsequently leads to economic loss (Mao et al., 2014).

Taurine is abundant in muscle tissue but has a low concentration in body fluids, such as serum and urine in pig (Huxtable, 1992; Wen et al., 2019), and it has varied biological and physiological functions, such as protective and inflammatory roles (Marcinkiewicz and Kontny, 2014; Wen et al., 2018, 2019). Taurine presents a high level in the small intestine (Huxtable and Lippincott, 1982). Several studies have shown that treatment with taurine has a protective effect on intestinal injuries owing to ischemia–reperfusion and lipopolysaccharides (Sukhotnik et al., 2016; Xiao et al., 2018), which is supported by the significant decrease in histological scores and apoptosis indices after ischemia–reperfusion in rats (Zhang et al., 2008). In dextran sulfate sodium-induced experimental colitis, taurine supplementation significantly alleviates diarrhea severity in C57BL/6 mice (Shimizu et al., 2009). In a model of potassium bromate-induced intestinal oxidative injury, taurine exerts an ameliorative effect by improving antioxidant defense and duodenum tissue integrity (Ahmad et al., 2015). However, some studies have shown that taurine impairs or has no beneficial effect on intestinal growth or on the mucosal structure of broiler chickens (Yuan and Wang, 2010; Huang et al., 2014). Excessive taurine supplementation (3%) was observed to have adverse effects on diarrhea index, villus height, and crypt depth (Liu et al., 2014). These results suggest that the dose of taurine supplementation used was not suitable in these experiments and that this aspect merits further research.

Normal intestinal function depends on the founding and conservation of a mucosal barrier, which is coated by a monolayer of intestinal epithelial cells. The intestinal mucosal barrier is indispensable for preventing intestinal injury owing to microbiota or undesirable substances (Guilloteau et al., 2010). However, the intestinal barrier is not fully developed at birth (Rouwet et al., 2002). The formation of tight junctions creates one of the major components of the intestinal barrier (Mitic et al., 2000). Occludin was the first identified tight junction protein, whereas claudins are the main proteins that contribute to the physiological and structural paracellular barrier function (Elkouby-Naor and Ben-Yosef, 2010). Researchers who have examined the intestinal barrier function mainly focus on the duodenum (Xiao et al., 2018), jejunum, and ileum (Sukhotnik et al., 2016), as well as on cell lines with H_2_O_2_-induced oxidative stress (Wang et al., 2012). In spite of this, there have been a few studies on the use of taurine in piglets. Liu et al. (2014) have reported the effect of taurine in the intestinal health of weaned pigs. Moreover, a few studies have collectively examined the duodenum, jejunum, and ileum to examine intestinal barrier function during oxidative stress.

Taking all of this into consideration, it is worthwhile to explore oxidative stress in the livestock industry. A previous study has found that the growth performance was negatively affected (such as reduced food intake, bodyweight, etc.) after diquat challenge (Yuan et al., 2017). Taurine supplementation (0.60%) abolished the depression of pig growth performance (unpublished data). Moreover, pigs are comparable to humans in terms of gastrointestinal tract structure and function; thus, these pig nutritional programming studies can provide reference for human nutrition studies (Guilloteau et al., 2010). In our previous study, we found that during oxidative stress, the protein expression of the taurine transporter is significantly increased, whereas it is ameliorated when taurine is added to the small intestines of piglets, and the concentration of taurine in blood is increased in a dose-dependent manner when fed diets contain different taurine content (Wen et al., 2019). However, whether taurine can alleviate the piglets’ intestinal oxidative stress through the restoration of the physiological function of the intestinal mucosa is not clear. Therefore, in this study, we hypothesized that taurine supplementation could alleviate diquat-induced intestinal damages; as such, we investigated the alleviative effects of taurine on piglets with diquat-induced intestinal oxidative stress.

## Materials and Methods

### Animals and Dieting

This study was conducted according to the Chinese Guidelines for Animal Care Welfare. Protocols were approved by the Committee on Animal Care of the Institute of Subtropical Agriculture, Chinese Academy of Sciences (ethical approval code: ISA-2017-012). Thirty-five weaned piglets were randomly allotted into five groups (*n* = 7). The control group (CON) was treated with a basic diet and intraperitoneal (ip) injection of isometric sterilized saline. The diquat group (DIQ) was treated with basic diet and ip administration of diquat; the low taurine group (LT) was treated with basic diet + 0.15% taurine and ip administration of diquat; the middle taurine group (MT) was treated with basic diet + 0.30% taurine and ip injection of diquat; and the high taurine group (HT) was treated with basic diet + 0.60% taurine and ip administration of diquat. According to a previous study, supplementing with 0.30% taurine increased the villus height, while 3% taurine supplementation reduced intestinal health of weaned piglets, such as higher diarrhea index, lower villus height, and deeper crypt depths (Liu et al., 2014). The saline or diquat treatments were conducted once at the beginning of the formal experiment. Diquat was purchased from Sigma (CAS Number 6358-62-2) and was dissolved in sterilized saline at a concentration of 9.6 mg/ml. Diquat would evoke vomit; therefore, diquat was administered by intraperitoneal injection. The injection volume was limited to 10 ml as described previously (Wen et al., 2019). The diets were isoenergetic, isonitrogenous, and were ensured to have met the nutritional requirements according to the National Research Council (2012). The content of taurine in diet was measured according to previous method (Wen et al., 2019). Feed composition is presented in Table 1.

**TABLE 1:** Ingredient and chemical composition of the experimental diet.

Items	Treatment			
	CON/DIQ	LT (0.15%)	MT (0.30%)	HT (0.60%)
**Ingredient composition (g/100 g)**				
Corn	64.15	64.23	64.38	64.61
Soybean meal	19.80	19.40	19.00	18.10
Soybean protein concentrate	5.00	5.00	5.00	5.00
Dried whey	4.30	4.30	4.30	4.30
Fish meal	4.00	4.00	4.00	4.00
Soya bean oil	0.35	0.50	0.60	0.90
Taurine	0.00	0.15	0.30	0.60
Lysine	0.36	0.37	0.37	0.41
Methionine	0.12	0.12	0.12	0.13
Threonine	0.09	0.10	0.10	0.11
Tryptophan	0.01	0.01	0.01	0.02
CaHPO_4_	0.00	0.00	0.00	0.00
Limestone	0.52	0.52	0.52	0.52
NaCl	0.30	0.30	0.30	0.30
1% Premix^a^	1.00	1.00	1.00	1.00
**Nutrient level (g/100 g)**				
Digestible energy(MJ/kg)^b^	14.59	14.59	14.58	14.57
Crude protein	20.11	20.12	20.11	20.12
SID lysine	1.24	1.23	1.23	1.24
SID methionine + cysteine	0.69	0.69	0.68	0.68
SID threonine	0.73	0.74	0.73	0.73
SID tryptophan	0.21	0.21	0.20	0.21
Total calcium	0.49	0.48	0.48	0.48
Total phosphorus	0.43	0.43	0.43	0.42
Digestible phosphorus	0.22	0.22	0.22	0.21
Taurine^c^	0.02	0.13	0.27	0.56

*^*a*^Supplied per kilogram of diet: CuSO_4_⋅5H_2_O, 19.8 mg; KI, 0.20 mg; FeSO_4_⋅7H_2_O, 400 mg; NaSeO_3_, 0.56 mg; ZnSO_4_⋅7H_2_O, 359 mg; MnSO_4_⋅H_2_O, 10.2 mg; vitamin K (menadione), 5 mg; vitamin B_1_, 2 mg; vitamin B_2_, 15 mg; vitamin B_12_, 30 μg; vitamin A, 5,400 IU; vitamin D_3_, 110 IU; vitamin E, 18 IU; choline chloride, 80 mg; antioxidants, 20 mg; fungicide, 100 mg. ^*b*^Calculated values, other values are measured. ^*c*^Measured values.*

### Sample Collection

After the feeding period (28 days), blood samples were collected from the overnight fasting piglets by vein puncture. To do this, pigs were electrically stunned (250 V, 0.5 A, 5–6 s) and bled by the exsanguination of the precaval vein. Blood samples were then centrifuged at 3,000 × *g* for 10 min, after which the supernatants were collected and stored at -80°C until the downstream analyses were performed. Intestinal mucosa samples were cleaned using phosphate-buffered saline (PBS) and were collected using a glass slide. All samples were placed into liquid N_2_ and stored at -80°C until further analysis.

### Serum Immunoglobulin

Serum immunoglobulin A (IgA) (CSB-E13234p), immunoglobulin G (IgG) (CSB-E06804p), and immunoglobulin M (IgM) (CSB-E06805p) levels were determined using an ELISA kit (Cusabio, Wuhan, China). All experiments were performed according to the manufacturer’s instructions. Briefly, we used a serum separator tube, and samples were allowed to clot for 2 h at room temperature before centrifugation for 15 min at 1,000 × *g*. It was recommended to dilute the serum with 2,000-fold dilution for test IgA and IgM and with 1,000-fold dilution for test IgG. This assay employs the competitive inhibition enzyme immunoassay technique. Then, we used the professional soft “Curve Expert” to make a standard curve. The concentration read from the standard curve were multiplied by the dilution factor.

### Analysis of Intestinal Morphology

Small intestine samples (duodenum, jejunum, ileum; 3 cm) were prepared for histological analysis; these were collected and immediately fixed in 4% neutral buffered formalin. Afterward, the samples were seated into paraffin blocks, and three cross-sections for each sample were cut into 6-μm-thick sections and stained with H&E. Villus height and crypt depth were measured from at least 12 well-oriented crypt–villus units by upright fluorescence microscopy using a model BX51 microscope (Olympus, Tokyo, Japan), as previously described (Wen et al., 2019).

### RNA Extraction and Real-Time RT-PCR

Reverse transcription and real-time quantitative PCR (RT-qPCR) were performed as previously described (Duan et al., 2017; Wen et al., 2019). TRIzol reagent (Invitrogen-Life Technologies, CA, United States) was used for total RNA extraction. Premixed Taq^TM^ DNA Polymerase (TaKaRa, Otsu, Japan) was used for PCR analysis. RT-PCR was performed by Roche LightCycler 480 II (Roche, Basel, Switzerland). The messenger RNA (mRNA) expression levels of the target genes were determined by the 2^–ΔΔCt^ method, using the following formula: [*ΔΔ*^Ct^ = (Ct_gene of interest_ – Ct_β–actin_)_treated_ – (Ct_gene of interest_ - Ct_β–actin_)_untreated_] (Li et al., 2015). β-Actin housekeeping gene was used as an internal control to normalize target gene transcript levels. Primers for the selected genes (Table 2) were designed using the Primer 5.0 software.

**TABLE 2:** Characteristics of the primers used for real-time PCR analysis.

Genes	Primer (from 5′ to 3′)	Product size (bp)	Accession no.
*Claudin-1*	F:CTCAATACAGGAGGGAAGCCA	91	NM-001244539.1
	R: ATATTTAAGGACCGCCCTCTCC		
*Occludin*	F: TCAGGTGCACCCTCCAGATT	167	NM-001163647.2
	R: TGTCGTTGCTGGGTGCATAA		
*ZO-1*	F:ACCATGTCTTGAAGCAGCCA	103	XM-021098896.1
	R: GCCTGTCTCTCCATGTACGG		
β*-actin*	F:CTGCGGCATCCACGAAACT	147	XM-021086047.1
	R: AGGGCCGTGATCTCCTTCTG		

*ZO-1, zonula occludens-1.*

### Western Blotting Analysis

Relative protein levels for the tight junction proteins ZO-1, claudin-1, and occludin in the small intestine were determined using Western blot (Duan et al., 2017; Wen et al., 2019). Protein content of intestine samples was detected by BCA Protein Assay Kit according to the manufacturer’s instructions (Beyotime, Shanghai, China). The resultant signals were obtained using the AlphaImager 2200 software (Alpha Innotech Corporation., San Leandro, CA, United States). The primary antibodies used in this experiment are ZO-1 (#21773-1-AP) at a dilution of 1:700, claudin-1 (#13050-1-AP) at a dilution of 1:500, and occludin (#66378-1-Ig) at a dilution of 1:1,000, and β-actin (#60008-1-Ig) at a dilution of 1:5,000, which were all purchased from Proteintech Group (Chicago, IL, United States).

### Statistical Analysis

The data were analyzed using one-way analysis of variance (ANOVA) and Duncan’s multiple range test (SAS 8.2). Data were expressed as mean ± SEM. Values in the same row marked with different letters are significantly different (*P* < 0.05).

## Results

### Serum Immunoglobin

**FIGURE 1:**
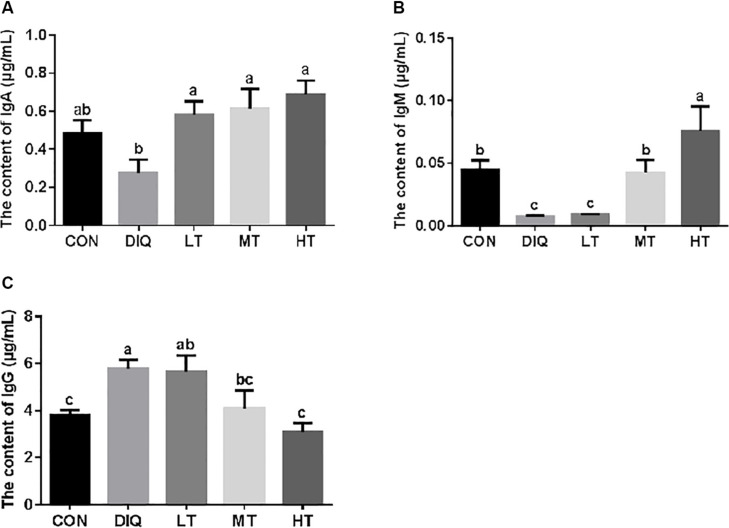
The concentration of serum immunoglobulin in diquat-induced oxidative stress with piglets. **(A)** The effect of taurine on serum IgA in diquat-induced oxidative stress of piglets; **(B)** the effect of taurine on serum IgM in diquat-induced oxidative stress of piglets; **(C)** the effect of taurine on serum IgG in diquat-induced oxidative stress of piglets. CON, control group; DIQ, diquat-treated group; LT, piglets supplemented with 0.15% taurine and treated with diquat; MT, piglets supplemented with 0.3% taurine and treated with diquat; HT, piglets supplemented with 0.6% taurine and treated with diquat. IgA, immunoglobulin A; IgM, immunoglobulin M; IgG, immunoglobulin G. Results were expressed as mean ± SEM, *n* = 7. ^a,b,c^Mean values with dissimilar letters were significantly different (*P* < 0.05).

The results of serum immunoglobin analysis are presented in Figure 1. Compared with CON, diquat-induced oxidative stress significantly decreased the IgM level and significantly increased IgG (*P* < 0.05). Compared with DIQ, taurine supplementation was able to restore the IgA levels with no dose effect. Compared with DIQ, IgM levels were significantly increased in MT and HT (*P* < 0.05), whereas IgG levels were significantly decreased in MT and HT (*P* < 0.05).

### Morphology

**FIGURE 2:**
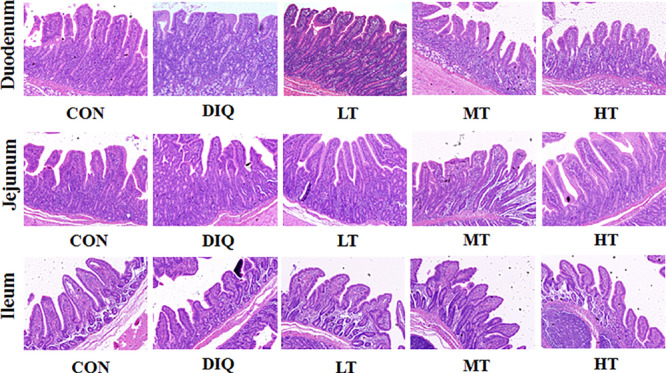
Effect of taurine on intestinal morphological structure in diquat-induced oxidative stress with weaned piglets (HE, × 200). CON, control group; DIQ, diquat-treated group; LT, piglets supplemented with 0.15% taurine and treated with diquat; MT, piglets supplemented with 0.3% taurine and treated with diquat; HT, piglets supplemented with 0.6% taurine and treated with diquat.

Figure 2 shows intestinal HE staining results (duodenum, jejunum, and ileum) of piglets with diquat-induced oxidative stress and taurine supplementation. Our results showed that supplementation with taurine could alleviate the intestinal injury to some extent.

**FIGURE 3:**
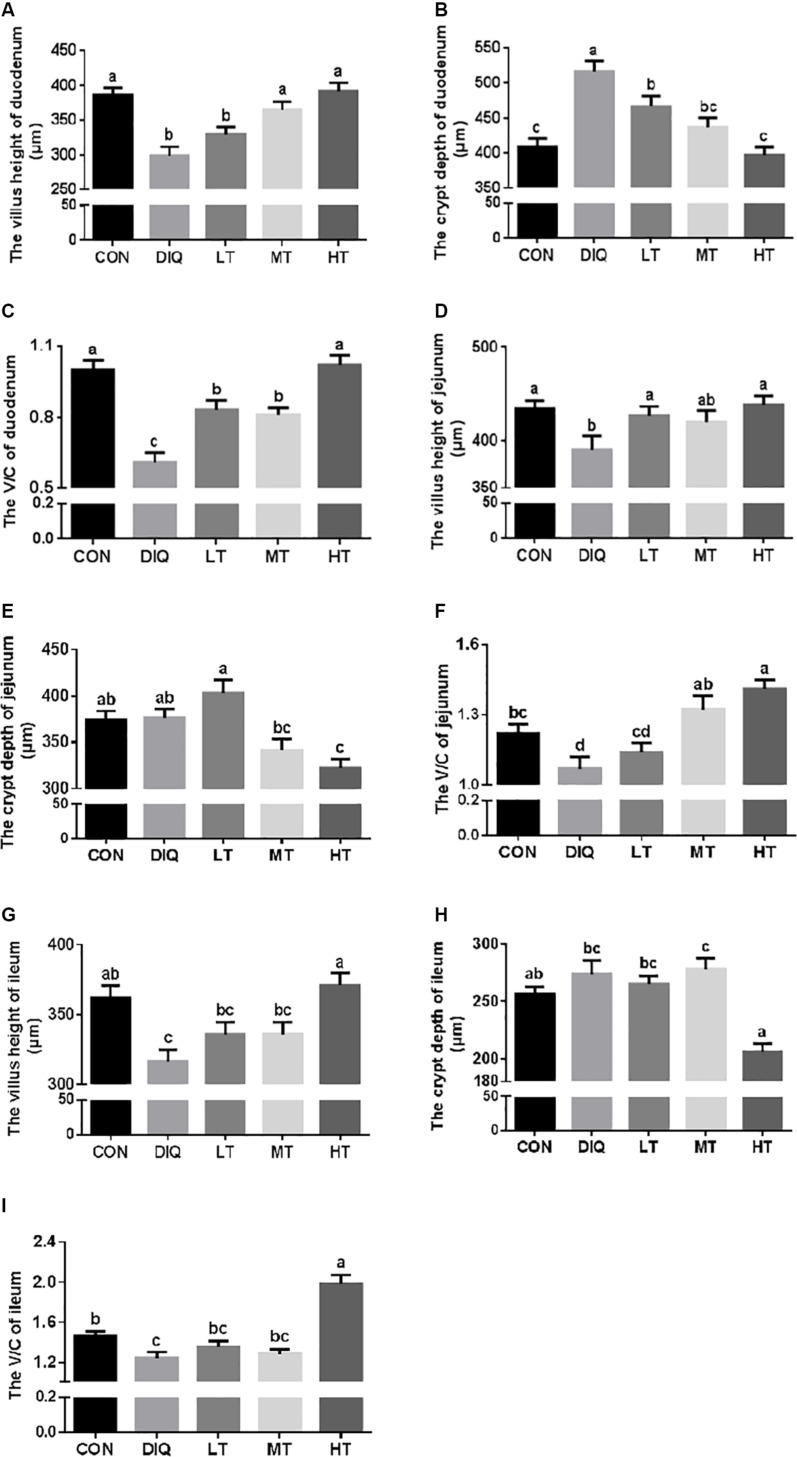
The small intestinal villus height and crypt depth with diquat-induced oxidative stress and taurine supplementation in piglets. **(A)** The effect of taurine on villus height of duodenum; **(B)** the effect of taurine on crypt depth of duodenum; **(C)** the effect of taurine on V/C of duodenum; **(D)** the effect of taurine on villus height of jejunum; **(E)** the effect of taurine on crypt depth of jejunum; **(F)** the effect of taurine on V/C of jejunum; **(G)** the effect of taurine on villus height of ileum; **(H)** the effect of taurine on crypt depth of ileum; **(I)** the effect of taurine on V/C of ileum. CON, control group; DIQ, diquat-treated group; LT, piglets supplemented with 0.15% taurine and treated with diquat; MT, piglets supplemented with 0.3% taurine and treated with diquat; HT, piglets supplemented with 0.6% taurine and treated with diquat. V/C, villus height/crypt depth. Results were expressed as mean ± SEM, *n* = 7. ^a,b,c,d^Mean values with dissimilar letters were significantly different (*P* < 0.05).

Figure 3 shows the measured values of intestinal villus height, crypt depth, and V/C ratio. Compared with CON, the villus height of the small intestine (duodenum, jejunum, and ileum) were significantly decreased in DIQ (*P* < 0.05); furthermore, we found that 0.60% taurine supplementation (HT) could restore the villus height of the small intestine. Compared with CON, the crypt depth of duodenum was significantly increased in DIQ (*P* < 0.05). Compared with DIQ, 0.60% taurine supplementation could effectively decrease crypt depth in the small intestine (*P* < 0.05). Compared with CON, V/C ratio was significantly decreased in DIQ, and compared with DIQ, 0.60% taurine supplementation was found to improve V/C ratio (*P* < 0.05).

### Tight Junction mRNA Expression

**FIGURE 4:**
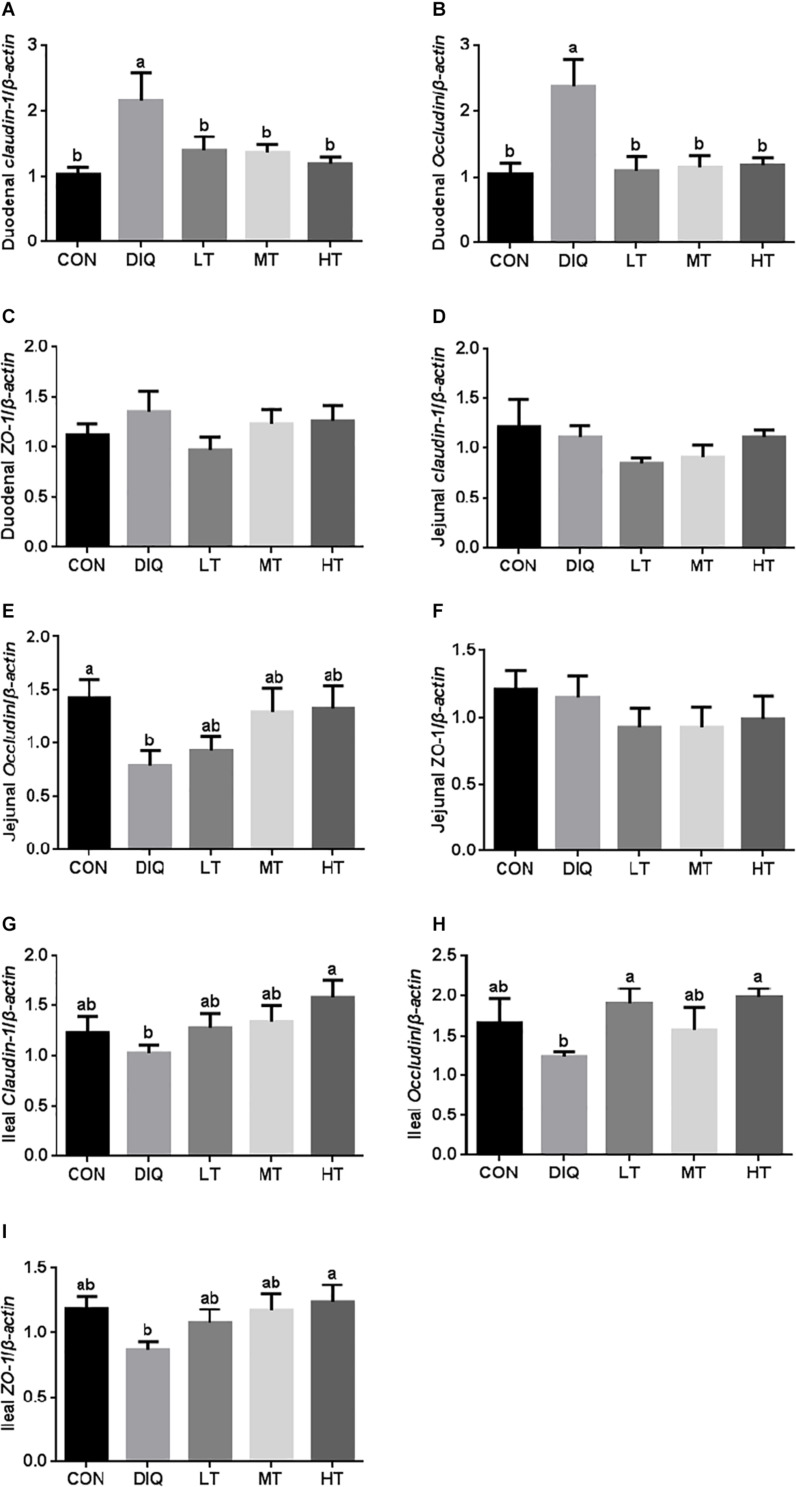
The small intestinal mucosa expression of tight junction-related genes in diquat-induced oxidative stress and taurine supplementation. **(A)** the effect of taurine on the messenger RNa (mRNA) expression of *claudin-1* in the duodenum; **(B)** the effect of taurine on the mRNA expression of *occludin* in the duodenum; **(C)** the effect of taurine on the mRNA expression of *ZO-1* in duodenum; **(D)** the effect of taurine on the mRNA expression of *claudin-1* in jejunum; **(E)** the effect of taurine on the mRNA expression of *occludin* in jejunum; **(F)** the effect of taurine on the mRNA expression of *ZO-1* in jejunum; **(G)** the effect of taurine on the mRNA expression of *claudin-1* in ileum; **(H)** the effect of taurine on the mRNA expression of *occludin* in ileum; **(I)** the effect of taurine on the mRNA expression of *ZO-1* in ileum. CON, control group; DIQ, diquat-treated group; LT, piglets supplemented with 0.15% taurine and treated with diquat; MT, piglets supplemented with 0.3% taurine and treated with diquat; HT, piglets supplemented with 0.6% taurine and treated with diquat. Results were expressed as mean ± SEM, *n* = 7. ^a,b^Mean values with dissimilar letters were significantly different (*P* < 0.05).

The mRNA expression levels of *claudin-1*, *occludin*, and *ZO-1* in the intestinal mucosa (duodenum, jejunum, and ileum) are presented in Figure 4. Compared with CON, the expression of *claudin-1* and *occludin* were significantly increased in DIQ in the duodenum; however, they had no difference in the taurine supplementation group (*P* < 0.05). Compared with CON, the expression of *occludin* was significantly decreased in DIQ in the jejunum (*P* < 0.05). Compared with DIQ, the expression of *claudin-1*, *occludin*, and *ZO-1* were significantly increased in the HT in the ileum (*P* < 0.05).

### Tight Junction Protein Expression

**FIGURE 5:**
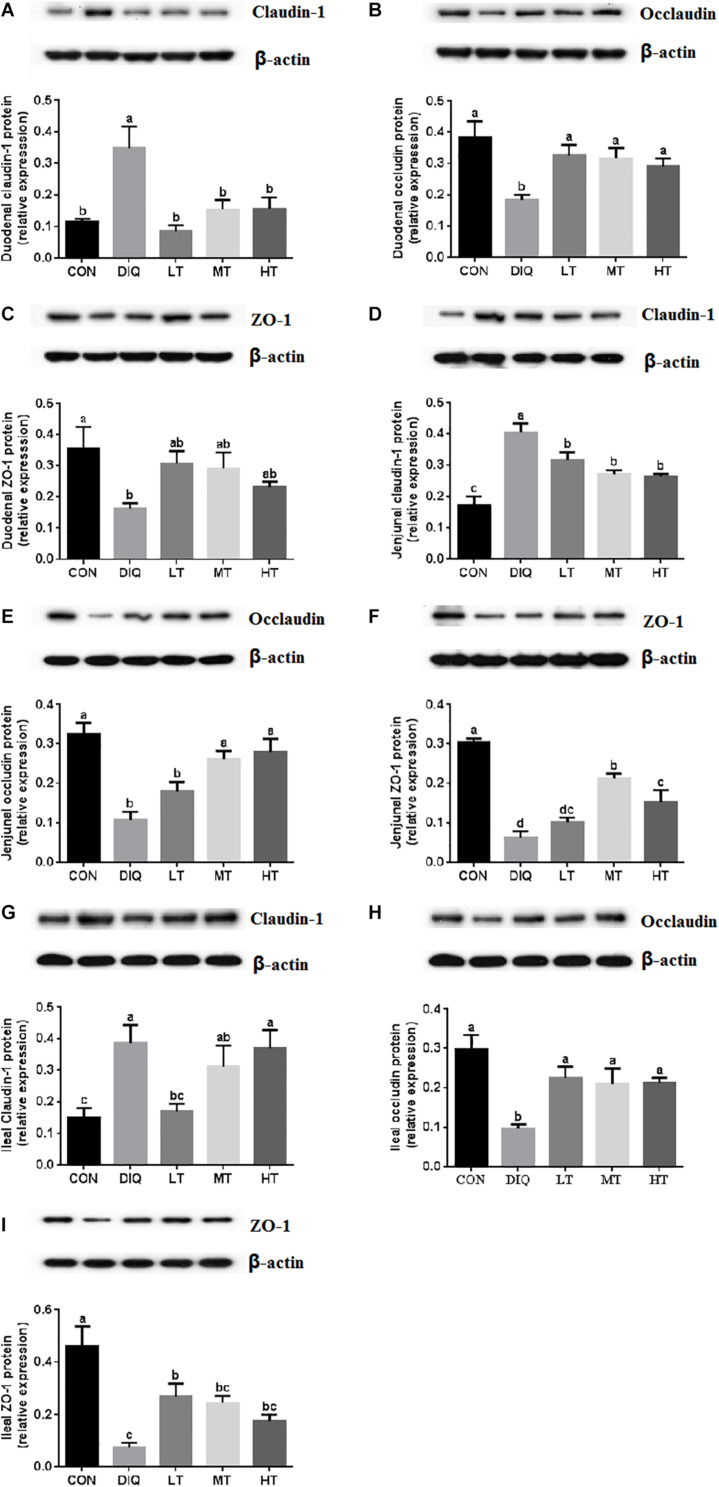
The small intestinal mucosa expression of tight junction-related proteins in diquat-induced oxidative stress and taurine supplementation. **(A)** The effect of taurine on the protein expression of claudin-1 in duodenum; **(B)** the effect of taurine on the protein expression of occludin in duodenum; **(C)** the effect of taurine on the protein expression of ZO-1 in duodenum; **(D)** the effect of taurine on the protein expression of claudin-1 in jejunum; **(E)** the effect of taurine on the protein expression of occludin in jejunum; **(F)** the effect of taurine on the protein expression of ZO-1 in jejunum; **(G)** the effect of taurine on the protein expression of claudin-1 in ileum; **(H)** the effect of taurine on the protein expression of occludin in ileum; **(I)** the effect of taurine on the protein expression of ZO-1 in ileum. CON, control group; DIQ, diquat-treated group; LT, piglets supplemented with 0.15% taurine and treated with diquat once time; MT, piglets supplemented with 0.3% taurine and treated with diquat once time; HT, piglets supplemented with 0.6% taurine and treated with diquat once time. Results were expressed as mean ± SEM, *n* = 7. ^a,b,c^Mean values with dissimilar letters were significantly different (*P* < 0.05).

The protein expression levels of claudin-1, occludin, and ZO-1 in the intestinal mucosa (duodenum, jejunum, and ileum) are presented in Figure 5. Compared with CON, the expression of claudin-1 was significantly upregulated in DIQ in the intestine (duodenum, jejunum, and ileum) (*P* < 0.05). Furthermore, compared with DIQ, taurine supplementation significantly downregulated the expression of claudin-1 in the duodenum, and jejunum (*P* < 0.05). Compared with CON, the expression of occludin and ZO-1 were significantly downregulated in DIQ in the intestine (duodenum, jejunum, and ileum) (*P* < 0.05). Compared with DIQ, taurine supplementation could significantly upregulate the expression of occludin in the intestine (duodenum, jejunum, and ileum), with the exception of LT in the jejunum (*P* < 0.05). Lastly, taurine supplementation was observed to elevate the ZO-1 expression in the intestine; however, the data were not significantly different (*P* > 0.05).

## Discussion

Weaning piglets frequently suffer from oxidative stress, which impairs growth and intestinal development (Hu et al., 2013; Yin et al., 2014; Cao et al., 2018). Taurine is known to exert a protective effect after damage (Wen et al., 2018). Our results indicated that oxidative stress triggers an aberrant immune response and has negative effects on intestinal morphology and tight junction barriers. Supplementation with taurine is an effective therapy for recovering, maintaining, or enhancing barrier function in diquat-induced intestinal injury.

Diquat is a toxin that affects cell survival and immune response (Deng et al., 2010; Nisar et al., 2015), whereas immunoglobulins play vital roles in mediating immune responses. Serum immunoglobulins include IgA, IgM, and IgG. The B and T cells were exposed to antigen and eventually swallowed and thus delivered to lymphoid cells; the B cells mature into plasma cells and secrete immunoglobulins (Conley and Delacroix, 1987). The immunoglobulin (IgA, IgG, and IgM) levels of the piglets were measured from the serum. IgG is the major serum immunoglobulin involved in mediating a protective inflammatory response (Kaneko et al., 2006); oxidative stress might play a role in the elevation of serum IgG (Nandakumar et al., 2008). IgA mediates a variety of protective functions and has both anti- and proinflammatory effects (Olas et al., 2005). Natural IgM has a primary function of protecting against a range of bacterial and fungal infections (Ehrenstein and Notley, 2010). Adult male workers exposed to insecticides have lower levels of IgG and IgM and higher levels of cytokines (Mecdad et al., 2011), which is consistent with our results. Immunoglobulin production is regulated by the nuclear factor kappa B (NF-κB) signaling pathway; it is concluded that oxidative-stress-induced activation of NF-κB signal pathway plays a possible role in the increase in serum immunoglobulin content (Nandakumar et al., 2008). It is suggested that oxidative stress alters the immune response, whereas taurine reverses these immunological abnormalities.

Physical barriers serve as the first line of defense against pathogenic agents in the external environment of the intestine (Pitman and Blumberg, 2000). Following infection with *Escherichia coli*, cytokine expression of IL-1β, IL-6, TNF-α, and IL-10 in the intestinal mucosa significantly increases (Ewaschuk et al., 2011), resulting in piglets having greater villous atrophy and intestinal morphology disruption (Yi et al., 2005). Weaning is related to villus atrophy and crypt hyperplasia (Montagne et al., 2007), and it is known that the small intestine crypt depth is increased starting from 3 to 7 days postweaning (Hedemann et al., 2003). On the other hand, dietary taurine has various effects on intestinal health. A study in chickens has shown that, when supplemented with 0.5 g/kg of taurine, the jejunum and ileum villi have impaired development and the relative height of the intestinal mucosa decreases (Yuan and Wang, 2010). Dietary supplementation of 0.3% taurine increases villus height, whereas 3% taurine increases diarrhea index and crypt depth and reduces villus height over that of the control (Liu et al., 2014). Thus, this study suggests that an appropriate dosage of taurine supplementation can benefit intestinal health. Supplementation with 60 mg/kg BW taurine significantly increased taurine concentration in the intestinal fluids of chickens (Xiao et al., 2018). Diquat significantly increases the content of reactive oxygen species, which could activate the mitophagy and proapoptotic-related signal pathway during intestinal injury (Zhou et al., 2006; Cao et al., 2018). In our study, we observed that oxidative stress impaired the development of the small intestinal morphology, shortened the villus height, decreased the ratio of V/C, and caused a negative effect on the crypt depth in the duodenum. Interestingly, we found that supplementation with 0.60% taurine increases villus height in the jejunum and ileum. Our results are consistent with those of a previous study that compared taurine-treated rats with ischemia–reperfusion animals, in which taurine caused a significant increase in villus height in the jejunum and ileum (Sukhotnik et al., 2016). Our data suggested that taurine potentially has a positive effect on intestinal damages induced by diquat.

Tight junctions form a cytoplasmic surface barrier between epithelial cells, play a central role in separating tissue compartments, and function in maintaining cellular polarity (Willott et al., 1993). In the animal model of diquat-induced oxidative stress, the concentration of TNF-α and IL-6 are significantly increased (Lu et al., 2010; Yuan et al., 2017), which was consistent with our previous results (unpublished data). Oxidative stress is a chronic inflammation, and it makes the intestine more susceptible to pathogens (Tian et al., 2017). Intestinal epithelial cells promote the appropriate immune response against enteric pathogens and modulate immune response at the intestinal barrier and promote the maintenance of intestinal orchestration (Peterson and Artis, 2014). Cytokines modulate tight junction protein expression in a protein-specific manner. Researchers have shown that TNF-α causes an increase in intestinal epithelial tight junction permeability (Ma et al., 2005; Ma and Ma, 2007). TNF-α induces a downregulation of claudin-1, occludin, and ZO-1 protein expression in Caco-2 cells (Ma et al., 2004). Proinflammatory cytokine IL-1β does not change ZO-1 protein expression but instead induces a progressive downregulation of occludin protein expression and an upregulation in claudin-1 protein expression in Caco-2 monolayers (Ma and Ma, 2007). In this study, we found that the protein expression of claudin-1 was significantly increased in DIQ in the small intestine, while the mRNA expression level of *claudin-1* was increased in the duodenum. Whether this phenomenon was induced by inflammatory cytokines still requires further investigation. Interestingly, our results showed that taurine supplementation downregulated claudin-1 protein expression in the duodenum and jejunum, low-dose taurine (0.15%) down-regulated claudin-1 protein expression, while middle- (0.30%) and high-dose (0.60%) taurine had no effect on claudin-1 protein expression in the ileum. Heat stress was found to increase occludin protein expression but had no effect on claudin-1 protein expression in the ileum of growing pigs (Pearce et al., 2013). Our results showed that oxidative stress had negative effects on occludin and ZO-1 protein expression in the small intestine, which is consistent with the results of a previous study (Cao et al., 2018). Furthermore, we found that the mRNA expression level of *occludin* increased, which may be related to a compensatory effect. Taurine supplementation could restore occludin and ZO-1 protein expression in the duodenum and ileum, and this did not exhibit dose dependence. However, a low dose (0.15%) of taurine had no effect on occludin and ZO-1 protein expression in the jejunum, which is consistent with jejunal occludin mRNA expression results. Lastly, the negative effect exerted on the intestinal tight barrier by oxidative stress was ameliorated by taurine.

Under oxidative stress condition, the content of taurine decreases in urine (Wen et al., 2019), suggesting that the reabsorption of taurine is improved. Lipopolysaccharide-induced inflammation has no effect on the concentration of taurine in serum and intestinal fluids (Xiao et al., 2018; Wen et al., 2019). However, the transportation of taurine is enhanced in the intestine. Cysteine dioxygenase (CDO) and cysteinesulfinate decarboxylase (CSD) are the two limited enzymes in taurine biosynthesis pathway of the liver, and its protein expression level is changed in a contrary way (Wen et al., 2018, 2019). Taking together, these results indicated that oxidative stress could lead to taurine redistribution in tissues and fluids. The synthesis of taurine was also changed in the liver.

## Conclusion

In conclusion, taurine could alleviate intestinal injury through the restoration of the intestinal morphology and tight junction barrier, with 0.60% taurine supplementation being a suitable dose. Our study provided a nutritional strategy for intestinal health. Further study is warranted to elucidate the intestinal permeability, inflammation, and immune responses that contribute to the intestinal pathologies that resulted from oxidative stress.

## Data Availability Statement

All relevant data is contained within the manuscript.

## Ethics Statement

The animal study was reviewed and approved by the Committee on Animal Care of the Institute of Subtropical Agriculture, Chinese Academy of Sciences (ethical approval code: ISA-2017-012).

## Author Contributions

CW contributed to the conceptualization, the methodology, the formal analysis, the investigation, the data curation, and the writing of the original draft. QG contributed to the methodology, the data curation, and the investigation. WW contributed to the methodology. YD contributed to the methodology and the investigation. LZ contributed to the investigation. JL and SH contributed to the supervision. WC contributed to the methodology. FL contributed to the resource, the review and editing aspect of the writing, the visualization, the supervision, and the project administration. All authors have read and approved the manuscript.

## Conflict of Interest

The authors declare that the research was conducted in the absence of any commercial or financial relationships that could be construed as a potential conflict of interest.

## Abbreviations

IgA: immunoglobulin A
IgG: immunoglobulin G
IgM: immunoglobulin M
V/C: villus height/crypt depth.

## References

[B1] Ahmad M. K. Khan A. A. Ali S. N. Mahmood R. (2015). Chemoprotective effect of taurine on potassium bromate-induced DNA damage, DNA-protein cross-linking and oxidative stress in rat intestine. *PLoS One* 10:e0119137 10.1371/journal.pone.0119137 PMC4352022 25748174

[B2] Boudry G. Peron V. Le Huerou-Luron I. Lalles J. P. Seve B. (2004). Weaning induces both transient and long-lasting modifications of absorptive, secretory, and barrier properties of piglet intestine. *J. Nutr.* 134 2256–2262. 10.1093/jn/134.9.2256 15333713

[B3] Campbell J. M. Crenshaw J. D. Polo J. (2013). The biological stress of early weaned piglets. *J. Anim. Sci. Biotechnol.* 4:19 10.1186/2049-1891-4-19 PMC3651348 23631414

[B4] Cao S. Wu H. Wang C. Zhang Q. Jiao L. Lin F. (2018). Diquat-induced oxidative stress increases intestinal permeability, impairs mitochondrial function, and triggers mitophagy in piglets. *J. Anim. Sci.* 96 1795–1805. 10.1093/jas/sky104 29562342 PMC6140957

[B5] Conley M. E. Delacroix D. L. (1987). Intravascular and mucosal immunoglobulin A: two separate but related systems of immune defense? *Ann. Intern. Med.* 106 892–899. 10.7326/0003-4819-106-6-892 3579073

[B6] Council N. R. (2012). *Nutrients Requirements of Swine.* Washington, DC: National Academy Press.

[B7] Deng Q. Xu J. Yu B. He J. Zhang K. Ding X. (2010). Effect of dietary tea polyphenols on growth performance and cell-mediated immune response of post-weaning piglets under oxidative stress. *Arch. Anim. Nutr.* 64 12–21. 10.1080/17450390903169138 20496858

[B8] Duan Y. Zeng L. Li F. Wang W. Li Y. Guo Q. (2017). Effect of branched-chain amino acid ratio on the proliferation, differentiation, and expression levels of key regulators involved in protein metabolism of myocytes. *Nutrition* 36 8–16. 10.1016/j.nut.2016.10.016 28336113

[B9] Ehrenstein M. R. Notley C. A. (2010). The importance of natural IgM: scavenger, protector and regulator. *Nat. Rev. Immunol.* 10 778–786. 10.1038/nri2849 20948548

[B10] Elkouby-Naor L. Ben-Yosef T. (2010). Functions of Claudin tight junction proteins and their complex interactions in various physiological systems. *Int. Rev. Cel. Mol. Biol.* 279 1–32. 10.1016/S1937-6448(10)79001-79008 20797675

[B11] Ewaschuk J. B. Murdoch G. K. Johnson I. R. Madsen K. L. Field C. J. (2011). Glutamine supplementation improves intestinal barrier function in a weaned piglet model of *Escherichia coli* infection. *Br. J. Nutr.* 106 870–877. 10.1017/S0007114511001152 21736826

[B12] Gu C. Qu H. Han L. Song X. Zhao L. Lu W. (2011). The effect of raw soybean on oxidative status of digestive organs in mice. *Int. J. Mol. Sci.* 12 8836–8845. 10.3390/ijms12128836 22272106 PMC3257103

[B13] Guilloteau P. Zabielski R. Hammon H. M. Metges C. C. (2010). Nutritional programming of gastrointestinal tract development. Is the pig a good model for man? *Nutr. Res. Rev.* 23 4–22. 10.1017/S0954422410000077 20500926

[B14] Guo Q. Li F. Duan Y. Wen C. Wang W. Zhang L. (2019). Oxidative stress, nutritional antioxidants and beyond. *Sci. China Life Sci.* [Online ahead of print] 10.1007/s11427-019-9591-5 31705360

[B15] Hedemann M. S. Hojsgaard S. Jensen B. B. (2003). Small intestinal morphology and activity of intestinal peptidases in piglets around weaning. *J. Anim. Physiol. Anim. Nutr.* 87 32–41. 10.1046/j.1439-0396.2003.00405.x 14511147

[B16] Hu C. Xiao K. Luan Z. Song J. (2013). Early weaning increases intestinal permeability, alters expression of cytokine and tight junction proteins, and activates mitogen-activated protein kinases in pigs. *J. Anim. Sci.* 91 1094–1101. 10.2527/jas.2012-5796 23230104

[B17] Huang C. Guo Y. Yuan J. (2014). Dietary taurine impairs intestinal growth and mucosal structure of broiler chickens by increasing toxic bile acid concentrations in the intestine. *Poult. Sci.* 93 1475–1483. 10.3382/ps.2013-03533 24879697

[B18] Huxtable R. J. (1992). Physiological actions of taurine. *Physiol. Rev.* 72 101–163. 10.1152/physrev.1992.72.1.101 1731369

[B19] Huxtable R. J. Lippincott S. E. (1982). Relative contribution of diet and biosynthesis to the taurine content of the adult rat. *Drug Nutr. Interact.* 1 153–168. 6926824

[B20] Kaneko Y. Nimmerjahn F. Ravetch J. V. (2006). Anti-inflammatory activity of immunoglobulin G resulting from Fc sialylation. *Science* 313 670–673. 10.1126/science.1129594 16888140

[B21] Li F. Duan Y. Li Y. Tang Y. Geng M. Oladele O. A. (2015). Effects of dietary n-6:n-3 PUFA ratio on fatty acid composition, free amino acid profile and gene expression of transporters in finishing pigs. *Br. J. Nutr.* 113 739–748. 10.1017/S0007114514004346 25704496

[B22] Liu Y. Mao X. Yu B. He J. Zheng P. Yu J. (2014). Excessive dietary taurine supplementation reduces growth performance, liver and intestinal health of weaned pigs. *Livest. Sci.* 168 109–119. 10.1016/j.livsci.2014.08.014

[B23] Lu T. Piao X. Zhang Q. Wang D. Piao X. Kim S. W. (2010). Protective effects of Forsythia suspensa extract against oxidative stress induced by diquat in rats. *Food Chem. Toxicol.* 48 764–770. 10.1016/j.fct.2009.12.018 20036301

[B24] Luo Z. Zhu W. Guo Q. Luo W. Zhang J. Xu W. (2016). Weaning induced hepatic oxidative stress, apoptosis, and aminotransferases through MAPK signaling pathways in piglets. *Oxid. Med. Cell Longev.* 2016:4768541 10.1155/2016/4768541 PMC5078666 27807471

[B25] Ma R. M. A. Ma T. Y. (2007). IL-1 causes an increase in intestinal epithelial tight junction permeability. *J. Immunol.* 178 4641–4649. 10.4049/jimmunol.178.7.4641 17372023 PMC3724221

[B26] Ma T. Y. Boivin M. A. Ye D. Pedram A. Said H. M. (2005). Mechanism of TNF-α modulation of Caco-2 intestinal epithelial tight junction barrier: role of myosin light-chain kinase protein expression. *Am. J. Physiol. Gastr. L* 288 G422–G430. 10.1152/ajpgi.00412.2004 15701621

[B27] Ma T. Y. Iwamoto G. K. Hoa N. T. Akotia V. Pedram A. Boivin M. A. (2004). TNF-alpha-induced increase in intestinal epithelial tight junction permeability requires NF-kappa B activation. *Am. J. Physiol. Gastr. L* 286 G367–G376. 10.1152/ajpgi.00173.2003 14766535

[B28] Mao X. Lv M. Yu B. He J. Zheng P. Yu J. (2014). The effect of dietary tryptophan levels on oxidative stress of liver induced by diquat in weaned piglets. *J. Anim. Sci. Biotechnol.* 5:49 10.1186/2049-1891-5-49 PMC4373006 25810902

[B29] Marcinkiewicz J. Kontny E. (2014). Taurine and inflammatory diseases. *Amino Acids* 46 7–20. 10.1007/s00726-012-1361-1364 22810731 PMC3894431

[B30] Mecdad A. A. Ahmed M. H. ElHalwagy M. E. A. Afify M. M. M. (2011). A study on oxidative stress biomarkers and immunomodulatory effects of pesticides in pesticide-sprayers. *Egyp. J. Forensic Sci.* 1 93–98. 10.1016/j.ejfs.2011.04.012

[B31] Mitic L. L. Van Itallie C. M. Anderson J. M. (2000). Molecular physiology and pathophysiology of tight junctions I. Tight junction structure and function: lessons from mutant animals and proteins. *Am. J. Physiol. Gastr. L* 279 G250–G254. 10.1152/ajpgi.2000.279.2.G250 10915631

[B32] Montagne L. Boudry G. Favier C. Le Huerou-Luron I. Lalles J. P. Seve B. (2007). Main intestinal markers associated with the changes in gut architecture and function in piglets after weaning. *Br. J. Nutr.* 97 45–57. 10.1017/S000711450720580X 17217559

[B33] Nandakumar D. N. Koner B. C. Vinayagamoorthi R. Nanda N. Negi V. S. Goswami K. (2008). Activation of NF-kappaB in lymphocytes and increase in serum immunoglobulin in hyperthyroidism: possible role of oxidative stress. *Immunobiology* 213 409–415. 10.1016/j.imbio.2007.10.005 18472049

[B34] Nisar R. Hanson P. S. He L. Taylor R. W. Blain P. G. Morris C. M. (2015). Diquat causes caspase-independent cell death in SH-SY5Y cells by production of ROS independently of mitochondria. *Arch. Toxicol.* 89 1811–1825. 10.1007/s00204-015-1453-1455 25693864 PMC4572080

[B35] Olas K. Butterweck H. Teschner W. Schwarz H. P. Reipert B. (2005). Immunomodulatory properties of human serum immunoglobulin A: anti-inflammatory and pro-inflammatory activities in human monocytes and peripheral blood mononuclear cells. *Clin. Exp. Immunol.* 140 478–490. 10.1111/j.1365-2249.2005.02779.x 15932509 PMC1809399

[B36] Pearce S. C. Mani V. Boddicker R. L. Johnson J. S. Weber T. E. Ross J. W. (2013). Heat stress reduces intestinal barrier integrity and favors intestinal glucose transport in growing pigs. *PLoS One* 8:e70215 10.1371/journal.pone.0070215 PMC3731365 23936392

[B37] Peterson L. W. Artis D. (2014). Intestinal epithelial cells: regulators of barrier function and immune homeostasis. *Nat. Rev. Immunol.* 14 141–153. 10.1038/nri3608 24566914

[B38] Pitman R. S. Blumberg R. S. (2000). First line of defense: the role of the intestinal epithelium as an active component of the mucosal immune system. *J. Gastroenterol.* 35 805–814. 10.1007/s005350070017 11085489

[B39] Pluske J. R. Hampson D. J. Williams I. H. (1997). Factors influencing the structure and function of the small intestine in the weaned pig: a review. *Livestock Product. Sci.* 51 215–236. 10.1016/s0301-6226(97)00057-52

[B40] Rouwet E. V. Heineman E. Buurman W. A. ter Riet G. Ramsay G. Blanco C. E. (2002). Intestinal permeability and carrier-mediated monosaccharide absorption in preterm neonates during the early postnatal period. *Pediatr. Res.* 51 64–70. 10.1203/00006450-200201000-200201012 11756641

[B41] Shimizu M. Zhao Z. Ishimoto Y. Satsu H. (2009). Dietary taurine attenuates dextran sulfate sodium (DSS)-induced experimental colitis in mice. *Adv. Exp. Med. Biol.* 643 265–271. 10.1007/978-0-387-75681-3_27 19239157

[B42] Sukhotnik I. Aranovich I. Ben Shahar Y. Bitterman N. Pollak Y. Berkowitz D. (2016). Effect of taurine on intestinal recovery following intestinal ischemia-reperfusion injury in a rat. *Pediatr. Surg. Int.* 32 161–168. 10.1007/s00383-015-3828-3823 26503339

[B43] Tian T. Wang Z. Zhang J. (2017). Pathomechanisms of oxidative stress in inflammatory bowel disease and potential antioxidant therapies. *Oxid. Med. Cell Longev.* 2017:4535194 10.1155/2017/4535194 PMC5506473 28744337

[B44] Wang N. Wang G. Hao J. Ma J. Wang Y. Jiang X. (2012). Curcumin Ameliorates hydrogen peroxide-induced epithelial barrier disruption by upregulating Heme Oxygenase-1 expression in human intestinal epithelial cells. *Digest. Dis. Sci.* 57 1792–1801. 10.1007/s10620-012-2094-2097 22392462

[B45] Wen C. Li F. Duan Y. Guo Q. Wang W. Zhang L. (2019). Dietary taurine regulates free amino acid profiles and taurine metabolism in piglets with diquat-induced oxidative stress. *J. Funct. Foods* 62:103569 10.1016/j.jff.2019.103569

[B46] Wen C. Li F. Zhang L. Duan Y. Guo Q. Wang W. (2018). Taurine is involved in energy metabolism in muscle, adipose tissue, and Liver. *Mol. Nutr. Food Res.* 63:e1800536 10.1002/mnfr.201800536 30251429

[B47] Willott E. Balda M. S. Fanning A. S. Jameson B. Van Itallie C. Anderson J. M. (1993). The tight junction protein ZO-1 is homologous to the Drosophila discs-large tumor suppressor protein of septate junctions. *Proc. Natl. Acad. Sci. U.S.A.* 90 7834–7838. 10.1073/pnas.90.16.7834 8395056 PMC47237

[B48] Xiao M. Mi Y. Liu L. Lv C. Zeng W. Zhang C. (2018). Taurine regulates mucosal barrier function to alleviate lipopolysaccharide-induced duodenal inflammation in chicken. *Amino Acids* 50 1637–1646. 10.1007/s00726-018-2631-2636 30132121

[B49] Yi G. F. Carroll J. A. Allee G. L. Gaines A. M. Kendall D. C. Usry J. L. (2005). Effect of glutamine and spray-dried plasma on growth performance, small intestinal morphology, and immune responses of *Escherichia coli* K88+-challenged weaned pigs. *J. Anim. Sci.* 83 634–643. 10.2527/2005.833634x 15705760

[B50] Yin J. Wu M. Xiao H. Ren W. Duan J. Yang G. (2014). development of an antioxidant system after early weaning in piglets. *J. Anim. Sci.* 92 612–619. 10.2527/jas2013-6986 24352957

[B51] Yuan D. Hussain T. Tan B. Liu Y. Ji P. Yin Y. (2017). The Evaluation of antioxidant and anti-inflammatory effects of *Eucommia ulmoides* flavones using diquat-challenged piglet models. *Oxid. Med. Cell Longev.* 2017:8140962 10.1155/2017/8140962 PMC5574320 28894511

[B52] Yuan J. Wang Z. (2010). Effect of taurine on intestinal morphology and utilisation of soy oil in chickens. *Br. Poult. Sci.* 51 540–545. 10.1080/00071668.2010.506984 20924849

[B53] Zhang F. Tong L. Qiao H. Dong X. Qiao G. Jiang H. (2008). Taurine attenuates multiple organ injury induced by intestinal ischemia reperfusion in rats. *J. Surg. Res.* 149 101–109. 10.1016/j.jss.2007.12.781 18639892

[B54] Zhou Y. Wang Q. Mark Evers B. Chung D. H. (2006). Oxidative stress-induced intestinal epithelial cell apoptosis is mediated by p38 MAPK. *Biochem. Biophys. Res. Commun.* 350 860–865. 10.1016/j.bbrc.2006.09.103 17034759 PMC2708970

